# Infections following temporary mechanical circulatory support in cardiogenic shock: Incidence, associations, and outcomes

**DOI:** 10.1016/j.jhlto.2026.100668

**Published:** 2026-08-14

**Authors:** Neil Shah, Gavin W. Hickey, Eun Jeong Kwak, Jianhui Zhu, Floyd Thoma, Raj Ramanan, Mark Schmidhofer, David Kaczorowski, Mohamed Abdullah, Holt Murray, Jonathan D. Wolfe, Ryan M. Rivosecchi

**Affiliations:** a Heart and Vascular Institute, University of Pittsburgh Medical Center (UPMC), Pittsburgh, PA, USA; b Division of Infectious Diseases, Department of Medicine, University of Pittsburgh School of Medicine, Pittsburgh, PA, USA; c Department of Critical Care Medicine, University of Pittsburgh Medical Center (UPMC), Pittsburgh, PA, USA; d Department of Cardiothoracic Surgery, University of Pittsburgh Medical Center (UPMC), Pittsburgh, PA, USA; e Department of Pharmacy, UPMC Presbyterian, Pittsburgh, PA, USA

**Keywords:** cardiogenic shock, temporary mechanical circulatory support, Impella, extracorporeal membrane oxygenation, nosocomial infection, outcomes

## Abstract

**Background:**

Infection complicates temporary mechanical circulatory support (tMCS) in cardiogenic shock (CS), but the contributions of device, severity, and timing remain unclear.

**Methods:**

Adults with CS supported by Impella, venoarterial extracorporeal membrane oxygenation (VA-ECMO), or both (ECPELLA) at a quaternary center (2021–2025) were studied retrospectively. Infection required clinician diagnosis, positive culture, and ≥5 days of targeted therapy. Cause-specific Cox and Fine-Gray competing-risks models assessed infection and a separate Cox model assessed 60-day mortality with infection as a time-varying covariate.

**Results:**

Among 297 patients, 111 (37.4%) developed a post-tMCS infection. Most first infections (75.7%) occurred within 14 days, with week-1 incidence over twice the average. Infected patients had longer hospital stays (35 vs 19 days, *p* < 0.001) and were less often discharged home (13.5% vs 31.2%, *p* < 0.001) but had similar in-hospital mortality (37.8% vs 37.6%, *p* = 0.972). Relative to Impella use alone, VA-ECMO use alone (cause-specific HR 2.70, 95% CI 1.63–4.48, *p* < 0.001) and ECPELLA use (HR 1.79, 95% CI 1.10–2.93, *p* = 0.020) were independently associated with infection (concordant on Fine-Gray analysis). Older age (HR 1.03/year, 95% CI 1.02–1.05, *p* < 0.001) and higher lactate at tMCS placement (HR 1.08 per mmol/L, 1.04–1.12, *p* < 0.001) were independently associated with 60-day mortality, while infection was not.

**Conclusions:**

Infection affected more than one-third of CS-tMCS patients, with peak risk in the first week. VA-ECMO support carried higher infection risk. Sixty-day mortality tracked older age and lactate at tMCS placement, but not device type. Infection was not associated with increased mortality but was associated with longer hospitalization and worse discharge disposition.

Cardiogenic shock (CS) carries mortality of 30–50% despite advances in medical and device-based therapy.^1^, ^2^ Temporary mechanical circulatory support (tMCS), including percutaneous microaxial flow pumps (Impella) and venoarterial extracorporeal membrane oxygenation (VA-ECMO), has become a cornerstone of CS management.^3^, ^4^ The Danish-German (DanGer) Shock trial showed that routine Impella CP use in ST-elevation myocardial infarction-related CS reduced 180-day mortality by 12.7% absolute, with durable benefit at long-term follow-up,^5^, ^6^ but at the cost of significantly higher rates of adverse events including sepsis, underscoring the complex risk-benefit profile of tMCS.^5^

Patients with CS are particularly susceptible to infection because of critical illness-associated immune dysregulation, compounded by invasive devices and prolonged mechanical ventilation.^7^, ^8^ Reported infection rates vary widely, from 26 to 64%, depending on definitions and populations.^9^, ^10^

Several knowledge gaps persist. The temporal pattern of infection risk is not well defined, and the relative contributions of device type and shock severity remain incompletely characterized. Existing studies also conflict on the infection-mortality association, with some reporting significant mortality effects^9^, ^11^ and others none.^12^ Resolving these questions is essential for guiding prevention and management.

We aimed to characterize infectious complications in patients with CS requiring tMCS, determining (1) the incidence, types, and timing of infection, (2) factors associated with infection development, (3) the microbiologic profile, and (4) how infection relates to clinical outcomes including length of stay (LOS), discharge disposition, and mortality.

## Methods

### Study design and setting

This single-center retrospective cohort study was conducted at the University of Pittsburgh Medical Center (UPMC) Presbyterian Hospital, a quaternary academic center with advanced heart failure and cardiac surgery programs. The institutional review board approved the study and waived informed consent given the retrospective design.

### Participants

Adults (≥18 years) admitted between January 2021 and December 2025 with CS who received Impella, VA-ECMO, or both (ECPELLA) during hospitalization were eligible, identified from institutional procedural databases with manual chart review. Patients were categorized by whether an infection developed after tMCS placement during the index admission (infection vs no infection).

### Device placement and antimicrobial prophylaxis

Impella placement occurred within the catheterization lab or operating room, while VA-ECMO cannulation occurred within the cardiothoracic ICU (CTICU) or operating room. Cannulation within the CTICU used full sterile precautions comparable to the operating room. No routine antimicrobial prophylaxis was used for tMCS device insertion or maintenance at our institution, and antimicrobials were given only to treat suspected or diagnosed infection.

### Variables and definitions

Infection was defined retrospectively, requiring (1) a clinician-documented infection diagnosis from an infectious disease service or critical care medicine team, (2) a positive culture from a clinically relevant source, and (3) a treatment of ≥5 consecutive days of targeted antimicrobial therapy. This approach was adopted given the retrospective nature of chart review and the variability in clinician documentation, and our infectious disease co-author (EJK) agreed with the method. The 5-day antimicrobial threshold was chosen to capture the full range of prescribing practices while still excluding brief empiric courses. *Clostridioides difficile* colitis was an exception to the positive culture requirement and was identified by positive stool toxin or PCR.

Each infection episode was classified per the 2024 International Society for Heart and Lung Transplantation (ISHLT) consensus definitions.^13^ MCS-specific infections comprised uncomplicated and complicated percutaneous lead infection, uncomplicated vascular cannulation site infection, complicated vascular cannula/sheath/graft infection, device-specific bloodstream infection, and device endocarditis. Non-MCS-specific infections comprised infective endocarditis of native or prosthetic valves, non-MCS bloodstream infections, sternal wound infections and mediastinitis, sepsis, and localized infections (eg, pneumonia, urinary tract infection, or *Clostridioides difficile* colitis).

Baseline characteristics comprised age, sex, SCAI stage at tMCS placement (≥C vs A-B), cardiac arrest at admission, device strategy (Impella alone, VA-ECMO alone, or ECPELLA), CS etiology (AMI vs non-AMI), diabetes, and serum lactate at tMCS placement. Antimicrobial use was characterized by use of any antibacterial or antifungal agents, the number of distinct agents, and exposure to any broad-spectrum antibiotics, with broad-spectrum classification per the Centers for Disease Control (CDC) National Healthcare Safety Network Antimicrobial Use Option.^14^ Antimicrobial treatment duration was defined as the number of days in which a patient had antimicrobial therapy administered.

### Outcomes

Outcomes included hospital and ICU length of stay, duration of tMCS support, any mechanical ventilation and ventilator-days, any hemodialysis/CRRT and dialysis-days, in-hospital and 60-day all-cause mortality, discharge disposition (home, skilled nursing/inpatient rehabilitation (SNF), transfer to other acute care), and bridge to durable left ventricular assist device (LVAD) or heart transplantation during the index admission. All-cause mortality was ascertained from the recorded date of death, which captures deaths occurring after hospital discharge.

### Timing and incidence-rate analysis

To characterize the temporal distribution of risk, time from tMCS placement to first infection was determined for each infected patient. Time-specific incidence rates were calculated as first infections per 100 patient-weeks at risk within sequential 7-day intervals, with patient-weeks obtained by summing each patient's hospitalized days per interval divided by 7, thereby adjusting for the declining at-risk denominator as patients were discharged or died.

### Statistical analysis

Continuous variables are presented as mean ± SD (Shapiro-Wilk-confirmed normality) or median (IQR) and compared with the *t*-test or Mann-Whitney *U* test. Categorical variables are reported as counts (%) and compared with the chi-square or Fisher's exact test. Comparisons were made between the infection and no-infection groups.

Factors associated with post-tMCS infection were evaluated within a competing-risks framework, because death, bridge to durable support, and discharge alive each preclude observing infection. Follow-up began at tMCS placement, with the event of interest being first infection and competing events being death/bridge/discharge. Analyses were truncated at 60 days, beyond which few patients remained at risk and cumulative-incidence estimates became unstable. We fit multivariable cause-specific Cox models (competing events censored) and, complementarily, Fine-Gray subdistribution models (competing events retained, quantifying effects on cumulative incidence). Device strategy was the exposure of interest, and the remaining baseline variables were entered simultaneously as covariates. To limit the covariate-to-event ratio and avoid overfitting, only a single pre-specified comorbidity (diabetes) was included, consistent with a comparable model.^15^ Proportional-hazards and proportional-subdistribution-hazards assumptions were checked with Schoenfeld residuals. Cumulative incidence functions (Aalen-Johansen) and Gray's test provided the unadjusted comparison across device strategies, whereas the cause-specific and Fine-Gray models gave covariate-adjusted estimates. Results are reported as cause-specific hazard ratios (HR) and subdistribution hazard ratios (sHR) with 95% CIs.

A separate multivariable Cox model evaluated factors associated with 60-day all-cause mortality. Sixty-day all-cause mortality was selected as a standard short-term endpoint in this population, capturing the acute support period. Patients bridged to durable LVAD or transplantation were excluded, as their trajectory and competing outcome differ fundamentally. Device strategy was modeled as three levels (Impella alone [reference], VA-ECMO alone, ECPELLA), with the remaining baseline variables entered as covariates. Infection was also added as a time-varying covariate to estimate its mortality association while avoiding immortal-time bias. Survival was compared across device strategies by Kaplan-Meier curves and log-rank test, and proportional hazards were checked with Schoenfeld residuals. Tests were two-sided (*p* < 0.05). All statistical analysis was run in R (survival, cmprsk packages), and figures were produced in Python (matplotlib). No a priori power calculation was performed.

## Results

### Study population and baseline characteristics

Of 297 patients, 111 (37.4%) developed at least one infection after tMCS placement and 186 (62.6%) remained infection-free. Baseline characteristics appear in Table 1. Groups were similar in age, sex, race, history of diabetes, SCAI stage ≥C at tMCS placement, and serum lactate at tMCS placement (median 3.7 vs 2.8 mmol/L, *p* = 0.683). Device strategy differed between groups (*p* = 0.016), with infected patients less often receiving Impella alone (38.7% vs 55.9%) and more often VA-ECMO alone (31.5% vs 22.0%) or ECPELLA (29.7% vs 22.0%).

**Table 1:** Baseline Characteristics of Patients by Infection Status

**Variable**	**No infection patients (*n* = 186)**	**Infection patients (*n* = 111)**	***p*-value**
*Demographics*			
Age, years, mean±SD	57±15	56±13	0.890
Female sex, *n* (%)	55 (29.6)	33 (29.7)	0.977
White race, *n* (%)	147 (79.0)	82 (73.9)	0.306
*Comorbidities*			
Diabetes mellitus, *n* (%)	46 (24.7)	35 (31.5)	0.203
*Clinical Characteristics*			
Cardiac arrest, *n* (%)	61 (32.8)	30 (27.0)	0.297
AMI etiology, *n* (%)	67 (36.0)	36 (32.4)	0.530
SCAI stage ≥C at MCS placement, *n* (%)	155 (83.3)	95 (85.6)	0.607
Lactate at MCS placement, mmol/L, median (IQR)	2.8 (1.5–7.0)	3.7 (1.4–7.3)	0.683
*Support Characteristics*			**0.016**
Impella alone, *n* (%)	104 (55.9)	43 (38.7)	
VA-ECMO alone, *n* (%)	41 (22.0)	35 (31.5)	
ECPELLA, *n* (%)	41 (22.0)	33 (29.7)	

MCS, mechanical circulatory support; SCAI, society for cardiovascular angiography and interventions; VA-ECMO, venoarterial extracorporeal membrane oxygenation.

Bold *p-values indicate statistical significance (p < 0.05).*

Device strategy comprises three mutually exclusive groups (Impella alone, VA-ECMO alone, ECPELLA) compared with a single Chi-square test.

### Clinical outcomes

Infected patients had longer hospital and ICU stays (Table 2). Median hospital stay nearly doubled (35 vs 19 days, *p* < 0.001) and ICU stay doubled (24 vs 12 days, *p* < 0.001), with longer tMCS duration (10 vs 8 days, *p* < 0.001), mechanical ventilation (10 vs 4 days, *p* < 0.001), and hemodialysis/CRRT (7 vs 3 days, *p* = 0.011). In-hospital and 60-day all-cause mortality were similar (37.8% vs 37.6%, *p* = 0.972%, and 33.3% vs 40.3%, *p* = 0.229), but discharge disposition differed: fewer were discharged home (13.5% vs 31.2%, *p* < 0.001) and more were discharged to rehabilitation or skilled nursing facilities (39.6% vs 26.3%, *p* = 0.017).

**Table 2:** Clinical Outcomes by Infection Status

**Outcome**	**No infection patients (*n* = 186)**	**Infection patients (*n* = 111)**	***p*-value**
*Length of Stay and Support*			
Hospital LOS, days, median (IQR)	19 (9**–**32)	35 (20**–**54)	**<0.001**
ICU LOS, days, median (IQR)	12 (6**–**21)	24 (14**–**37)	**<0.001**
Total tMCS duration, days, median (IQR)	8 (4**–**14)	10 (6**–**24)	**<0.001**
Any mechanical ventilation, *n* (%)	160 (86.0)	109 (98.2)	**<0.001**
Ventilator days, median (IQR)	4 (2**–**8)	10 (5**–**23)	**<0.001**
Any hemodialysis or CRRT, *n* (%)	35 (18.8)	37 (33.3)	**0.005**
Hemodialysis or CRRT days, median (IQR)	3 (1**–**9)	7 (4**–**14)	**0.011**
*Mortality*			
In-hospital mortality, *n* (%)	70 (37.6)	42 (37.8)	0.972
60-day all-cause mortality, *n* (%)^a^	75 (40.3)	37 (33.3)	0.229
*Discharge Disposition*			
Home, *n* (%)	58 (31.2)	15 (13.5)	**<0.001**
Rehabilitation or SNF, *n* (%)	49 (26.3)	44 (39.6)	**0.017**
Transfer, *n* (%)	9 (4.8)	10 (9.0)	0.155
Bridge to LVAD or transplant, *n* (%)	38 (20.4)	30 (27.0)	0.191

LOS, length of stay; ICU LOS, intensive care unit length of stay; SNF, skilled nursing facility; CRRT, continuous renal replacement therapy; LVAD, left ventricular assist device; tMCS, temporary mechanical circulatory support.

Bold *p*-values indicate statistical significance (*p* < 0.05).

a 60-day all-cause mortality was lower than in-hospital mortality for infection patients because some patients were still hospitalized 60 days after tMCS placement but later died while still in the hospital.

### Timing of first infection

**Figure 1:**
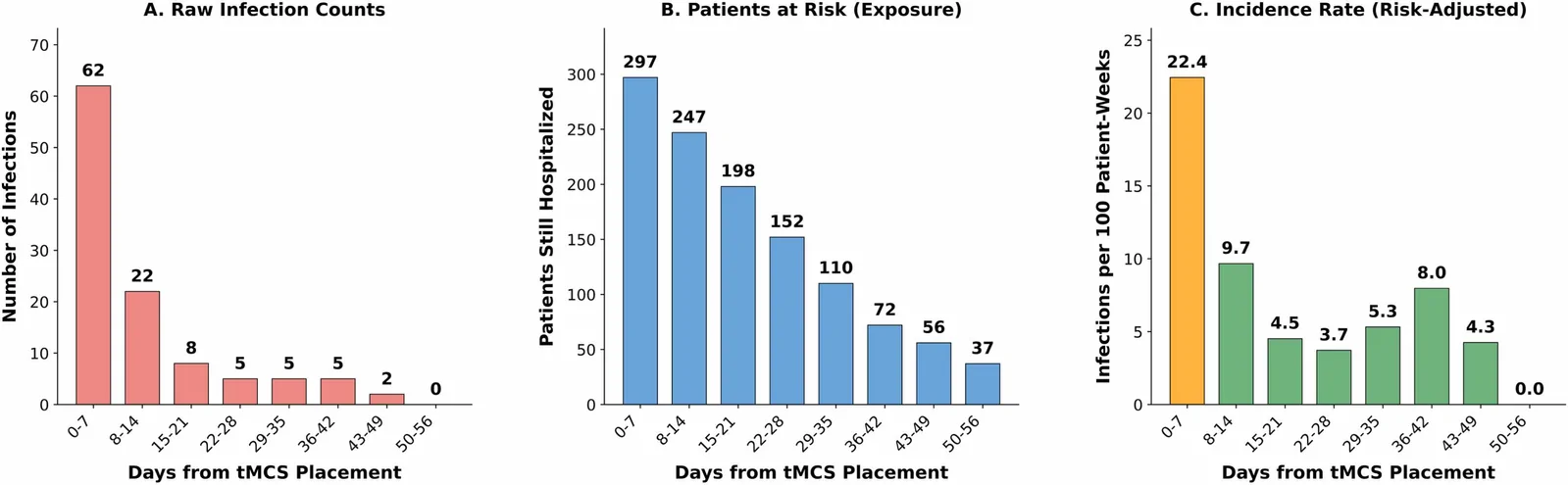
Timing of infection following temporary mechanical circulatory support (tMCS) placement. (A) Raw counts of first infections by 7-day interval. (B) Number of patients at risk during each interval. (C) Time-specific incidence rate (first infections per 100 patient-weeks at risk), accounting for declining at-risk denominator. The yellow bar in panel C highlights the week with the highest incidence rate. The week-1 incidence rate (22.4 per 100 patient-weeks) was more than double the overall cohort average (10.4 per 100 patient-weeks) and approximately five-fold higher than the rate in weeks 3-4, confirming a period of genuinely elevated infection risk. The figure displays first infections occurring within 56 days of tMCS placement (*n* = 109). Two additional first infections occurred later (at days 64 and 83) and are not shown, accounting for the difference between the bar totals and the 111 infected patients in the full cohort.

Median time to first infection was 7 days (IQR 3–13). Most first infections occurred early: 55.9% (62/111) within 7 days and 75.7% (84/111) within 14 days. Time-specific incidence-rate analysis confirmed genuinely elevated early risk rather than merely more patients still hospitalized (Figure 1): the week-1 rate (days 0–7) was 22.4 first infections per 100 patient-weeks, more than double the cohort average of 10.4 and roughly five times the weeks 3–4 rate.

### Infection characteristics and microbiology

Of the total 297 patients, 111 (37.4%) developed at least one post-tMCS infection (Table 3). Non-MCS-specific infections predominated, with 104 patients (35.0%) developing a non-MCS-specific infection and only 14 (4.7%) developing an MCS-specific infection. Overall infection incidence differed by device strategy, with infection occurring in 46.1% of patients supported with VA-ECMO alone and 44.6% with ECPELLA, compared with 29.3% of patients receiving Impella alone. Patient-level incidences of each infection type, organized by device strategy, are detailed in Table 3. Since some patients developed more than one type of infection, subtype proportions do not sum to the overall incidence. Additionally, some patients developed the same type of infection more than once. Because patient-level proportions count each patient only once per infection type, we also characterized individual episodes to better capture infection burden.

**Table 3:** Infection Incidence by Device Strategy

**Infection category/subtype (ISHLT 2024)**	**Impella-only (*N* = 147)**	**VA-ECMO-only (*N* = 76)**	**ECPELLA (*N* = 74)**	**All (*N* = 297)**
*Patients in device group (N)*	147	76	74	297
*Developed any infection*	43 (29.3%)	35 (46.1%)	33 (44.6%)	111 (37.4%)
*MCS-specific infection (any)*	6 (4.1%)	3 (3.9%)	5 (6.8%)	14 (4.7%)
Uncomplicated vascular cannulation site infection	2 (1.4%)	1 (1.3%)	-	3 (1.0%)
Complicated vascular cannula/sheath/graft infection	2 (1.4%)	-	2 (2.7%)	4 (1.3%)
Device-specific bloodstream infection	2 (1.4%)	2 (2.6%)	4 (5.4%)	8 (2.7%)
*Non-MCS-specific infection (any)*	39 (26.5%)	34 (44.7%)	31 (41.9%)	104 (35.0%)
Pneumonia	26 (17.7%)	24 (31.6%)	21 (28.4%)	71 (23.9%)
Non-MCS bloodstream infections	10 (6.8%)	6 (7.9%)	5 (6.8%)	21 (7.1%)
Infective endocarditis of native or prosthetic valves	1 (0.7%)	-	-	1 (0.3%)
Sepsis	4 (2.7%)	4 (5.3%)	5 (6.8%)	13 (4.4%)
Urinary tract infection	5 (3.4%)	-	3 (4.1%)	8 (2.7%)
Clostridioides difficile colitis	2 (1.4%)	1 (1.3%)	3 (4.1%)	6 (2.0%)
Sternal wound infections and mediastinitis	-	2 (2.6%)	3 (4.1%)	5 (1.7%)
Other localized infection	2 (1.4%)	-	1 (1.4%)	3 (1.0%)

ISHLT, International Society for Heart and Lung Transplantation; MCS, mechanical circulatory support.

Values are number of patients (% of all patients in specific device strategy group). Infections were classified per the 2024 ISHLT consensus definitions. Since a patient could develop more than one infection type, subtype percentages do not sum to the “Developed any infection” row. “-” indicates no patients.

Among the 111 infection patients, 147 distinct clinician-diagnosed infection episodes were documented. A total of 183 organism isolates were recovered, indicating some episodes were polymicrobial. Microbiologic patterns tracked infection type more than device strategy (Table 4). Pneumonia, the most common episode in every device group, was predominantly gram-negative (75% of pneumonia isolates), while non-MCS bloodstream infections were mostly gram-positive (61%). Across device groups, the organism mix was broadly similar and gram-negative-predominant, though ECPELLA carried the greatest *Pseudomonas* and overall gram-negative burden and VA-ECMO accounted for most *Candida* isolates. Resistant organisms (MRSA, methicillin-resistant *S. epidermidis*, vancomycin-resistant *Enterococcus*, and ESBL-producing or multidrug-resistant gram-negatives) occurred across all three groups.

**Table 4:** Infection Episodes by Device Strategy and ISHLT 2024 Subtype: Source Organisms and Treatment Duration

**Infection type (ISHLT 2024)**	**Episodes, *n* (% of group total)**	**Source organisms (isolates, *n*)**	**Median treatment duration, days (IQR)**
*Impella-only (n = 57 episodes)*			
MCS-SPECIFIC INFECTIONS			
Uncomplicated vascular cannulation site infection	2 (3.5%)	Pseudomonas aeruginosa (1); Staphylococcus epidermidis (MSSE) (1)	10.5 (10–11)
Complicated vascular cannula/sheath/graft infection	2 (3.5%)	Enterobacter cloacae (1); Pseudomonas aeruginosa (1); Pseudomonas aeruginosa (MDR) (1)	30.5 (25–36)
Device-specific bloodstream infection	2 (3.5%)	Staphylococcus epidermidis (MSSE) (1); Staphylococcus lugdunensis (1)	19 (17–22)
NON-MCS-SPECIFIC INFECTIONS			
Pneumonia	26 (45.6%)	Serratia marcescens (7); Klebsiella pneumoniae (4); Enterobacter cloacae (3); Escherichia coli (3); Pseudomonas aeruginosa (3); Staphylococcus aureus (MRSA) (3); Staphylococcus aureus (MSSA) (3); Acinetobacter baumannii (1); Burkholderia cepacia complex (1); Citrobacter freundii (1); Haemophilus influenzae (1); Moraxella catarrhalis (1); Staphylococcus epidermidis (MSSE) (1)	7 (7–14)
Non-MCS bloodstream infections	11 (19.3%)	Enterococcus faecium (VRE) (3); Candida spp. (2); Enterococcus faecalis (2); Klebsiella pneumoniae (ESBL) (2); Mixta (Pantoea) calida (2); Staphylococcus aureus (MRSA) (2); Klebsiella oxytoca/Raoultella spp. (1); Staphylococcus epidermidis (MRSE) (1); Staphylococcus epidermidis (MSSE) (1); Streptococcus mitis group (1)	14 (9–27)
Infective endocarditis of native or prosthetic valves	1 (1.8%)	Enterococcus faecium (VRE) (1); Klebsiella pneumoniae (ESBL) (1); Mixta (Pantoea) calida (1); Staphylococcus aureus (MRSA) (1)	51 (51–51)
Sepsis	4 (7.0%)	Enterobacter cloacae (1); Enterobacter cloacae (MDR) (1); Escherichia coli (1); Klebsiella oxytoca/Raoultella spp. (1); Staphylococcus aureus (MSSA) (1); Stenotrophomonas maltophilia (1)	21 (19–22)
Urinary tract infection	5 (8.8%)	Escherichia coli (2); Staphylococcus epidermidis (MSSE) (2); Citrobacter koseri (1)	10 (7–13)
Clostridioides difficile colitis	2 (3.5%)	Clostridioides difficile (2)	14 (14–14)
Other localized infection	2 (3.5%)	Enterococcus faecalis (1); Escherichia coli (1); Morganella morganii (1); Staphylococcus aureus (MRSA) (1)	21 (18–25)
*VA-ECMO-only (n = 42 episodes)*			
MCS-SPECIFIC INFECTIONS			
Uncomplicated vascular cannulation site infection	1 (2.4%)	Staphylococcus epidermidis (MSSE) (1)	16 (16–16)
Device-specific bloodstream infection	3 (7.1%)	Enterococcus faecalis (1); Enterococcus faecium (VRE) (1); Klebsiella aerogenes (1)	21 (18–32)
NON-MCS-SPECIFIC INFECTIONS			
Pneumonia	25 (59.5%)	Staphylococcus aureus (MSSA) (6); Klebsiella pneumoniae (4); Pseudomonas aeruginosa (4); Enterobacter cloacae (2); Klebsiella aerogenes (2); Streptococcus pneumoniae (2); Achromobacter xylosoxidans (1); Acinetobacter baumannii (1); Escherichia coli (1); Klebsiella oxytoca/Raoultella spp. (1); Pseudomonas fluorescens (1); Serratia marcescens (1); Staphylococcus aureus (MRSA) (1); Staphylococcus hominis (1); Stenotrophomonas maltophilia (1)	7 (7–10)
Non-MCS bloodstream infections	6 (14.3%)	Candida albicans (1); Enterococcus faecium (VRE) (1); Pseudomonas aeruginosa (1); Staphylococcus epidermidis (MRSE) (1); Staphylococcus epidermidis (MSSE) (1); Staphylococcus saccharolyticus (1)	14 (9–19)
Sepsis	4 (9.5%)	Acinetobacter baumannii (2); Serratia marcescens (2); Acinetobacter bereziniae (1); Candida spp. (1); Citrobacter freundii (1); Staphylococcus aureus (MSSA) (1)	24.5 (22–27)
Clostridioides difficile colitis	1 (2.4%)	Clostridioides difficile (1)	14 (14–14)
Sternal wound infections and mediastinitis	2 (4.8%)	Candida spp. (2)	49 (39–60)
*ECPELLA (n = 48 episodes)*			
MCS-SPECIFIC INFECTIONS			
Complicated vascular cannula/sheath/graft infection	2 (4.2%)	Escherichia coli (1); Klebsiella variicola (1); Staphylococcus lugdunensis (1)	17.5 (16–19)
Device-specific bloodstream infection	4 (8.3%)	Candida spp. (1); Klebsiella variicola (1); Staphylococcus aureus (MSSA) (1); Staphylococcus lugdunensis (1)	29.5 (27–31)
NON-MCS-SPECIFIC INFECTIONS			
Pneumonia	22 (45.8%)	Klebsiella pneumoniae (4); Klebsiella oxytoca/Raoultella spp. (3); Pseudomonas aeruginosa (3); Escherichia coli (2); Klebsiella pneumoniae (ESBL) (2); Staphylococcus aureus (MRSA) (2); Staphylococcus aureus (MSSA) (2); Aspergillus (1); Burkholderia cepacia complex (1); Citrobacter freundii (1); Citrobacter koseri (1); Enterobacter cloacae (1); Enterobacterales (unspeciated) (1); Morganella morganii (1); Pseudomonas aeruginosa (MDR) (1); Serratia marcescens (1)	11 (7–14)
Non-MCS bloodstream infections	5 (10.4%)	Enterobacter cloacae (1); Klebsiella spp. (1); Staphylococcus aureus (MRSA) (1); Staphylococcus aureus (MSSA) (1); Staphylococcus epidermidis (MRSE) (1)	15 (14–40)
Sepsis	5 (10.4%)	Enterococcus faecalis (1); Escherichia coli (1); Haemophilus influenzae (1); Proteus vulgaris group (1); Pseudomonas aeruginosa (MDR) (1); Serratia marcescens (1); Staphylococcus aureus (MRSA) (1)	25 (10–29)
Urinary tract infection	3 (6.3%)	Escherichia coli (1); Klebsiella oxytoca/Raoultella spp. (1); Klebsiella variicola (1)	5 (5–5)
Clostridioides difficile colitis	3 (6.3%)	Clostridioides difficile (3)	19 (17–25)
Sternal wound infections and mediastinitis	3 (6.3%)	Candida spp. (1); Pseudomonas aeruginosa (1); Pseudomonas aeruginosa (MDR) (1)	42 (32–45)
Other localized infection	1 (2.1%)	Pseudomonas aeruginosa (1)	27 (27–27)

Episodes are grouped within each device strategy (Impella-only, VA-ECMO-only, ECPELLA). Each row is one infection episode; a patient may contribute more than one episode. Source organisms are isolates cultured from episodes of that type, with the number of isolates in parentheses (an episode may yield more than one organism). Median treatment duration is the documented antimicrobial treatment duration per episode in days, with interquartile range. “/” within an organism name denotes a single co-reported isolate.

### Antimicrobial utilization

Antimicrobial exposure was substantially higher among infected patients. Nearly all received broad-spectrum antibiotics (99.1% vs 74.2%, *p* < 0.001) and antifungal exposure was more frequent (27.0% vs 7.5%, *p* < 0.001). Infected patients received more distinct antibacterial agents (median 4 vs 2, *p* < 0.01) and longer antimicrobial treatment duration (median 14 vs 6 days, *p* < 0.001). Median antimicrobial treatment duration was similar between device groups for each infection type (Table 4).

### Factors associated with infection occurrence

**Figure 2:**
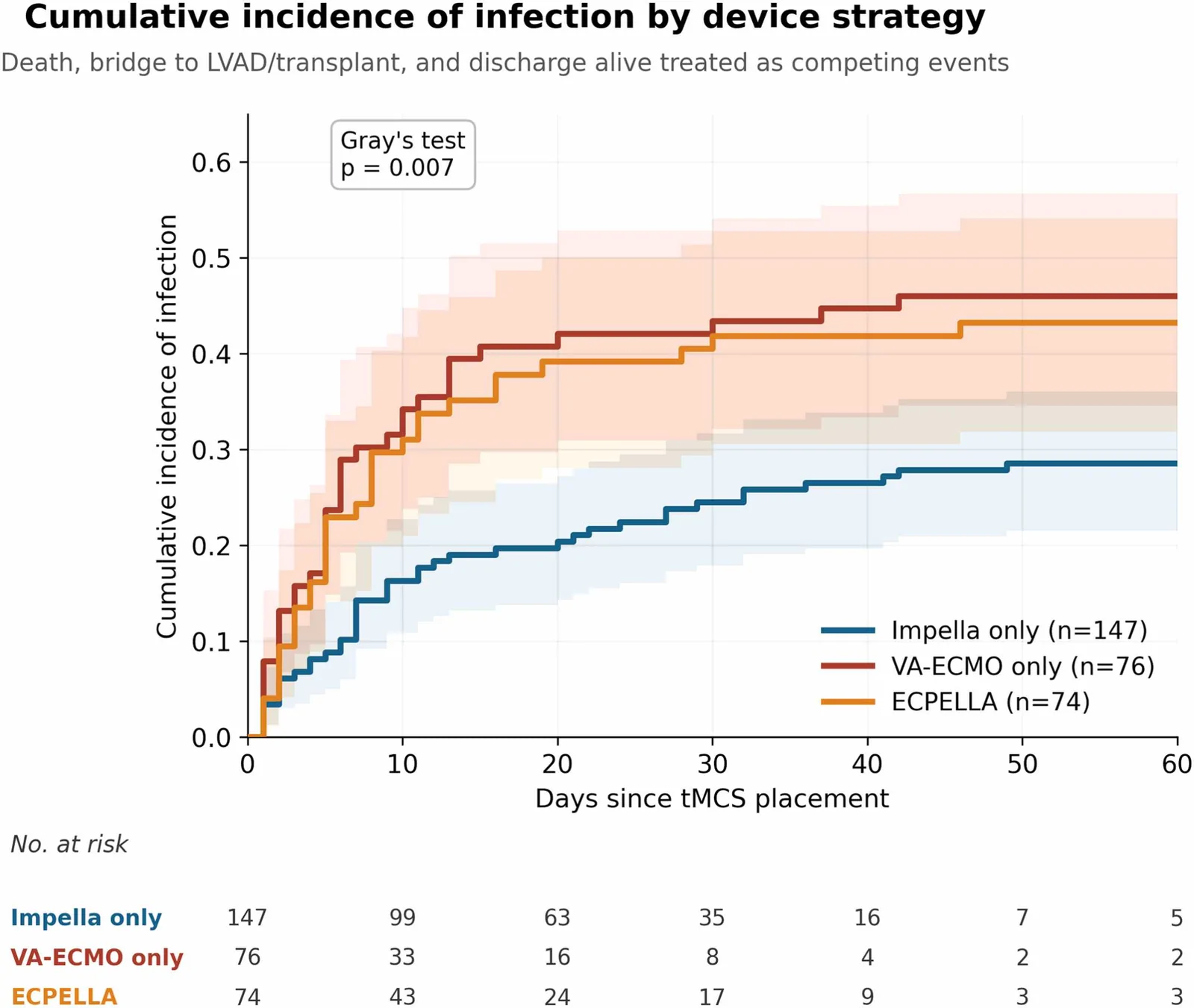
Cumulative incidence of infection after temporary mechanical circulatory support (tMCS) by device strategy, with death, bridge to durable left ventricular assist device or transplantation, and discharge alive treated as competing events. Cumulative incidence functions were estimated with the Aalen-Johansen estimator over a 60-day horizon; shaded bands denote 95% confidence intervals and the number at risk is shown below the x-axis. By day 60 the cumulative incidence of infection was 46% with VA-ECMO alone, 43% with ECPELLA, and 29% with Impella alone (Gray's test *p* = 0.007). ECPELLA, concurrent Impella and VA-ECMO support; VA-ECMO, venoarterial extracorporeal membrane oxygenation.

In the competing-risks analysis, the cumulative incidence of infection differed across device strategies (Figure 2; Gray's test *p* = 0.007), reaching 46% with VA-ECMO alone and 43% with ECPELLA versus 29% with Impella alone by day 60. Device strategy was the only factor associated with infection (Table 5). Relative to Impella alone, the cause-specific hazard was higher for VA-ECMO alone (HR 2.70, 95% CI 1.63–4.48, *p* < 0.001) and ECPELLA (HR 1.79, 95% CI 1.10–2.93, *p* = 0.020). Fine-Gray models were concordant (sHR 2.44, 1.44–4.12, *p* = 0.001; and 2.05, 1.25–3.36, *p* = 0.004), indicating a genuinely higher cumulative incidence. The two VA-ECMO-containing strategies did not differ (cause-specific HR 1.51, 0.91–2.49, *p* = 0.11). Proportional-hazards assumptions were satisfied.

**Table 5:** Factors Associated With Infection After Temporary Mechanical Circulatory Support in Multivariable Competing-Risks Models (60-Day Horizon)

**Term**	**Adjusted cause-specific HR (95% CI)**	***p*-value**	**Adjusted fine-gray sHR (95% CI)**	***p*-value**
VA-ECMO only	**2.70** (1.63–4.48)	**<0.001**	**2.44** (1.44–4.12)	**0.001**
ECPELLA	**1.79** (1.10–2.93)	**0.020**	**2.05** (1.25–3.36)	**0.004**
Age (per year)	1.01 (1.00–1.03)	0.071	1.01 (0.99–1.02)	0.31
Female sex	1.03 (0.67–1.57)	0.90	0.99 (0.63–1.55)	0.96
Cardiac arrest	0.85 (0.54–1.33)	0.48	0.76 (0.48–1.19)	0.23
SCAI stage ≥ C	1.01 (0.59–1.74)	0.96	1.10 (0.61–1.97)	0.75
AMI etiology	1.10 (0.72–1.69)	0.67	1.03 (0.66–1.61)	0.88
Diabetes mellitus	1.32 (0.87–2.00)	0.19	1.31 (0.87–1.97)	0.20
Lactate (per mmol/L)	1.01 (0.97–1.05)	0.78	0.98 (0.94–1.01)	0.17

SCAI, society for cardiovascular angiography and interventions; VA-ECMO, venoarterial extracorporeal membrane oxygenation.

Reference group: Impella only. *n* = 297, with 109 infections within 60 days. Both models were multivariable: every estimate is mutually adjusted for all covariates shown (device strategy, age, sex, cardiac arrest, SCAI stage ≥ C, AMI etiology, diabetes mellitus, and serum lactate). Bold indicates *p* < 0.05.

### Factors associated with mortality

**Figure 3:**
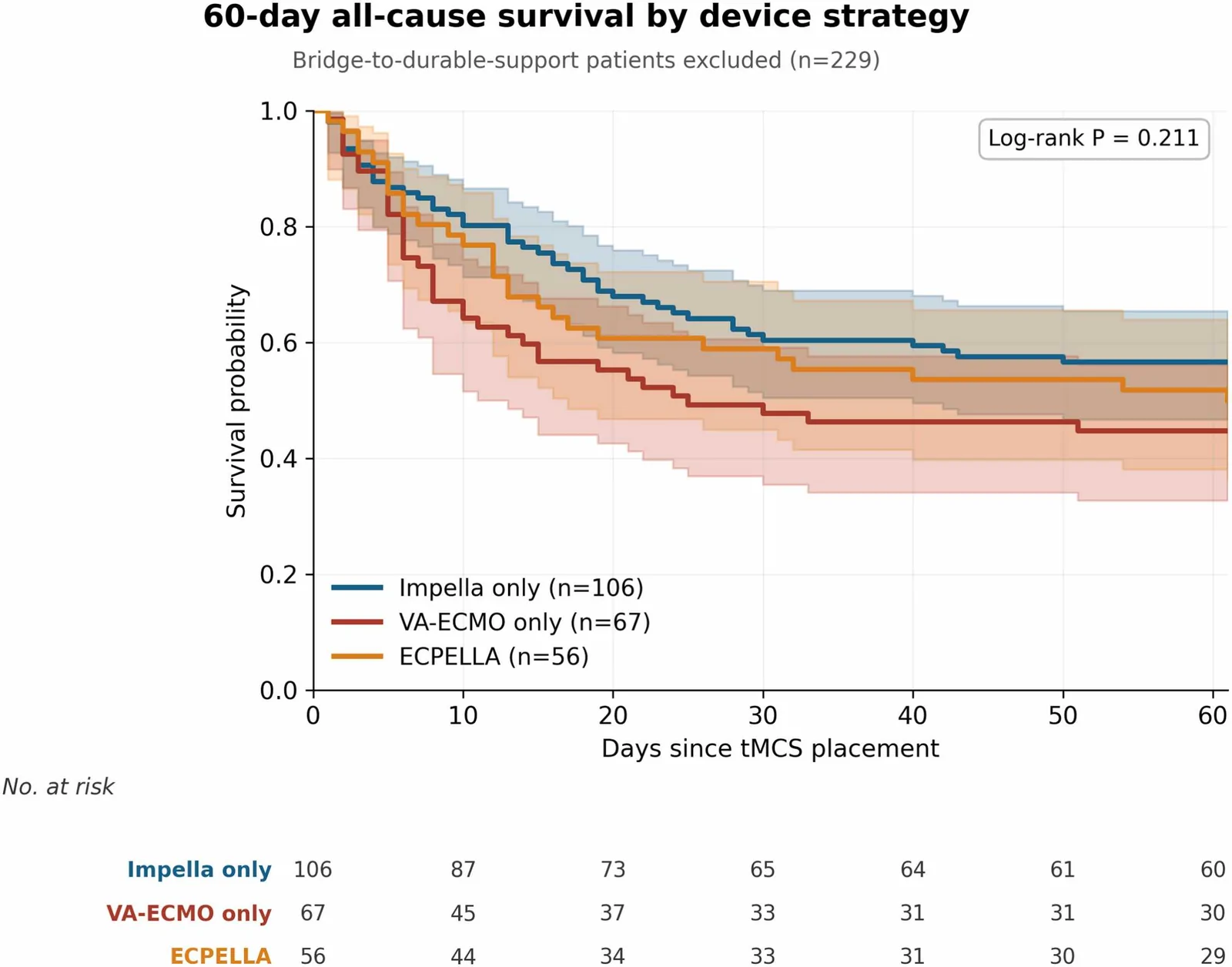
Kaplan-Meier curves of 60-day all-cause survival by device strategy (Impella alone, VA-ECMO alone, or concurrent Impella plus VA-ECMO [ECPELLA]) among patients not bridged to durable support (*n* = 229; 111 deaths within 60 days). Death was ascertained from the recorded date of death, which captures deaths after discharge. Shaded bands are 95% confidence intervals and the number at risk is shown below the x-axis. By day 60, survival was 57% with Impella alone, 52% with ECPELLA, and 45% with VA-ECMO alone, without a significant difference across device strategies (log-rank *p* = 0.211). ECPELLA, concurrent Impella and VA-ECMO support; VA-ECMO, venoarterial extracorporeal membrane oxygenation.

After excluding the 68 bridged patients, a Cox model for 60-day all-cause mortality in the remaining 229 (111 deaths) identified older age (HR 1.03/year, 95% CI 1.02–1.05, *p* < 0.001) and higher serum lactate at tMCS placement (HR 1.08 per mmol/L, 1.04–1.12, *p* < 0.001) as the factors independently associated with mortality (Table 6). Relative to Impella alone, neither VA-ECMO alone (HR 1.53, 0.91–2.56, *p* = 0.11) nor ECPELLA (HR 1.10, 0.65–1.86, *p* = 0.72) was independently associated with mortality. Infection, modeled as a time-varying covariate, was not associated with mortality (HR 1.24, 0.80–1.92, *p* = 0.34). On Kaplan-Meier analysis, 60-day survival was 57% (Impella alone), 52% (ECPELLA), and 45% (VA-ECMO alone), without a significant difference (log-rank *p* = 0.211; Figure 3). Consistent with the model, in-hospital mortality was nearly identical between infected and uninfected patients (37.8% vs 37.6%, *p* = 0.972).

**Table 6:** Factors Associated With 60-Day All-Cause Mortality After Temporary Mechanical Circulatory Support (Multivariable Cox Proportional-Hazards Model With Infection as a Time-Varying Covariate)

**Term**	**Adjusted hazard ratio (95% CI)**	***p*-value**
VA-ECMO only	1.53 (0.91–2.56)	0.11
ECPELLA	1.10 (0.65–1.86)	0.72
Age (per year)	**1.03** (1.02–1.05)	**<0.001**
Lactate (per mmol/L)	**1.08** (1.04–1.12)	**<0.001**
Cardiac arrest at admission	1.22 (0.81–1.85)	0.34
AMI etiology	0.72 (0.47–1.10)	0.13
Diabetes mellitus	0.70 (0.45–1.10)	0.13
SCAI stage ≥ C	1.04 (0.61–1.76)	0.89
Female sex	0.85 (0.56–1.32)	0.48
Infection (time-varying)	1.24 (0.80–1.92)	0.34

SCAI, society for cardiovascular angiography and interventions; VA-ECMO, venoarterial extracorporeal membrane oxygenation.

Reference group: Impella only. *n* = 229 after exclusion of 68 patients bridged to durable support; 111 deaths within 60 days. Estimates are adjusted hazard ratios from a single multivariable model: every hazard ratio is mutually adjusted for all covariates shown (device strategy, age, serum lactate, cardiac arrest, AMI etiology, diabetes mellitus, SCAI stage ≥ C, sex, and infection). Infection was modeled as a time-varying covariate, and lactate was measured at tMCS placement. Bold indicates *p* < 0.05.

## Discussion

In this single-center cohort of 297 patients with CS requiring tMCS, four principal findings emerged. First, infection developed in more than one-third of patients (37.4%). Second, relative to Impella use alone, both VA-ECMO use alone (HR 2.70, Fine-Gray sHR 2.44) and ECPELLA (HR 1.79, sHR 2.05) were independently associated with higher infection incidence, and device strategy was the only factor associated with infection. Third, the first week after tMCS placement was a period of genuinely elevated infection risk. Fourth, infection was not found to be associated with greater mortality. Only older age and higher serum lactate at tMCS placement, and not device strategy, were independently associated with 60-day all-cause mortality.

The 37.4% overall infection rate aligns with prior reports. INTERMACS data identified infection as the most common adverse event in mechanically supported patients,^16^ Schmidt and colleagues reported 64% nosocomial infection in VA-ECMO-supported CS,^9^ and a meta-analysis of 4,733 ECMO patients reported a pooled 26% incidence (95% CI 14–38%).^10^ Our intermediate rate likely reflects a rigorous definition requiring clinician diagnosis, microbiologic confirmation, and ≥5 days of targeted antimicrobials.

Device strategy was the only factor independently associated with infection. Both VA-ECMO-containing strategies carried higher risk and did not differ from each other, identifying VA-ECMO exposure rather than the specific configuration as the marker of risk. The concordance of cause-specific and Fine-Gray estimates also indicates a genuinely higher cumulative infection incidence. This is biologically plausible as VA-ECMO requires large-bore cannulation and prolonged circuit exposure and has been associated with immune alterations, including myeloid-derived suppressor-cell expansion and immunosuppressive cytokine shifts, early after initiation.^17^ Consistent with this, the week-1 incidence in the overall cohort was more than double the cohort average, suggesting the period of greatest immune vulnerability coincides with greatest infection risk. These findings support intensified prevention (catheter care, ventilator-associated pneumonia bundles, and early ventilator liberation) during the first 1–2 weeks of support.^18^, ^19^

Whereas device strategy shaped infection risk, it did not shape the microbiology. The organism profile reflected the healthcare-associated nature of the infection episodes and tracked infection type more than device strategy. Pneumonia, which dominated in every device group, was largely gram-negative, whereas non-MCS bloodstream infections were predominantly gram-positive. Resistant organisms, including methicillin-resistant *Staphylococcus aureus*, vancomycin-resistant *Enterococcus*, and ESBL-producing or multidrug-resistant gram-negatives, were distributed across all device groups. Combined with the greater number of days on antimicrobial treatment in infected patients (median 14 vs 6 days), this profile underscores the importance of local antibiograms for therapy^18^ and of antimicrobial stewardship in this population.

Applying the ISHLT 2024 definitions clarified that the overwhelming majority of infections were non-MCS-specific rather than device-related. Only 16 of 147 episodes (10.9%) were MCS-specific, and no episodes of percutaneous lead infection or device endocarditis occurred. Since Impella and VA-ECMO are accessed through vascular cannulas rather than a tunneled driveline, the lead-infection category did not apply to these devices. The absence of device endocarditis reflected active evaluation rather than incomplete ascertainment, as patients with sustained bloodstream or fungal infection and clinical suspicion of endocarditis were assessed with echocardiography. The frequency of non-MCS-specific infection fits the short-term, peripherally cannulated nature of temporary MCS and the ISHLT observation that non-MCS-specific infections are more commonly seen with acute support.^13^ It suggests VA-ECMO raises risk less through the cannula and more through prolonged mechanical ventilation, extended ICU exposure, and critical-illness immune dysregulation compounded by the extracorporeal circuit.^8^

Despite its high prevalence, predictable timing, and substantial healthcare burden, infection did not appear to increase mortality. Modeled as a time-varying covariate, infection was not associated with 60-day mortality (HR 1.24, 95% CI 0.80–1.92, *p* = 0.34), consistent with Nair and colleagues^12^ but contrasting with Aslam and colleagues, who identified early bloodstream infection as an independent predictor.^11^ However, because our analysis was not powered to exclude a moderate effect, this null likely reflects a failure to detect rather than a true absence of association. In our results, mortality more closely tracked baseline patient condition. Older age and higher serum lactate at tMCS placement were independently associated with mortality.

Although we did not detect an association between infection and mortality, functional recovery differed strikingly. Only 13.5% of infected patients were discharged home versus 31.2% (*p* < 0.001), with more discharged to rehabilitation or skilled nursing facilities (39.6% vs 26.3%, *p* = 0.017). Since infected patients spent more time in the ICU, there was likely a greater risk of ICU-acquired weakness and post-intensive care syndrome (physical, cognitive, and psychological impairments following critical illness), which may in turn have led to a lower likelihood of home discharge and greater discharge to rehabilitation or long-term care.^20^, ^21^, ^22^ These associations suggest that, in this population, infection may be more closely linked to post-critical-illness disability than to mortality, with potential implications for resource allocation and post-discharge planning.

## Limitations

Several limitations warrant acknowledgment. The retrospective single-center design may limit generalizability. Our infection definition, requiring clinician diagnosis in documentation, microbiologic confirmation, and ≥5 days of targeted antimicrobials, attempts to minimize misclassification due to the nature of retrospective review, but is more restrictive than surveillance criteria such as the CDC/NHSN definitions.^23^ By requiring a positive culture, we possibly underestimate true infection burden, as clinicians may strongly suspect infection (new infiltrates, fever, hemodynamic deterioration) and begin antibiotics without obtaining cultures. Conversely, colonization events may yield positive cultures, inflating the rate of infection and over-attributing respiratory and urinary diagnoses. Additionally, since respiratory cultures are obtained more readily in intubated patients, the infection group having more days on ventilation would likely have resulted in differential surveillance. Finally, the study lacked an a priori power calculation and the mortality analysis was likely underpowered, so our finding of no association between infection and mortality may reflect a type II error rather than true absence of effect.

## Conclusions

Infection affects more than one-third of patients with CS receiving tMCS. VA-ECMO exposure was independently associated with infection, whereas older age and higher serum lactate at tMCS placement, not device strategy, were independently associated with 60-day all-cause mortality. Infection risk is highest during the first week of support, with respiratory and bloodstream infections predominating and notable antimicrobial resistance. Infection was not independently associated with increased mortality but was associated with prolonged hospitalization and worse discharge disposition. These findings support intensified infection-prevention efforts during early tMCS support, particularly in VA-ECMO patients.

## IRB statement

This study was approved by the University of Pittsburgh Institutional Review Board, with the requirement for informed consent waived given the retrospective nature of the study.

## CRediT authorship contribution statement

**Neil Shah:** Writing - original draft, Writing - review & editing, Visualization, Investigation, Methodology, Validation, Formal analysis, Conceptualization, **Gavin W. Hickey:** Writing - review & editing, Investigation, Methodology, Validation, Supervision, Conceptualization, **Eun Jeong Kwak:** Writing - review & editing, Investigation, Methodology, Validation, Supervision, Conceptualization, **Jianhui Zhu:** Writing - review & editing, Methodology, Data curation, Formal analysis, **Floyd Thoma:** Writing - review & editing, Methodology, Data curation, Conceptualization, **Raj Ramanan:** Writing - review & editing, Investigation, Methodology, Validation, Conceptualization, **Mark Schmidhofer:** Writing - review & editing, Investigation, Methodology, Validation, Conceptualization, **David Kaczorowski:** Writing - review & editing, Investigation, Methodology, Validation, Conceptualization, **Mohamed Abdullah:** Writing - review & editing, Investigation, Methodology, Validation, Conceptualization, **Holt Murray:** Writing - review & editing, Investigation, Methodology, Validation, Conceptualization, **Jonathan D. Wolfe:** Writing - review & editing, Investigation, Methodology, Validation, Conceptualization, **Ryan M. Rivosecchi:** Writing - review & editing, Investigation, Methodology, Validation, Supervision, Conceptualization

## Declaration of Competing Interest

The authors declare the following financial interests/personal relationships which may be considered as potential competing interests: Consulting Fees from Abiomed/Johnson and Johnson - Gavin W. Hickey. Consulting Fees from Johnson & Johnson MedTech and TransMedics - David Kaczorowski. If there are other authors, they declare that they have no known competing financial interests or personal relationships that could have appeared to influence the work reported in this paper.

## Data Availability

Data are available upon reasonable request from the corresponding author, consistent with institutional policy.
